# Epigenetic aging in patients diagnosed with coronary artery disease: results of the LipidCardio study

**DOI:** 10.1186/s13148-023-01434-8

**Published:** 2023-01-31

**Authors:** Verena Laura Banszerus, Maximilian König, Ulf Landmesser, Valentin Max Vetter, Ilja Demuth

**Affiliations:** 1 Department of Endocrinology and Metabolic Diseases (Including Division of Lipid Metabolism), Biology of Aging Working Group, Charité – Universitätsmedizin Berlin, Corporate Member of Freie Universität Berlin and Humboldt-Universität zu Berlin, Augustenburger Platz 1, 13353 Berlin, Germany; 2 Department of Cardiology, Charité Universitätsmedizin Berlin, Campus Benjamin Franklin (CBF), Berlin, Germany; 3 Berlin Institute of Health (BIH), Deutsches Zentrum Für Herzkreislaufforschung (DZHK), Partner Site Berlin, Berlin, Germany; 4 BCRT - Berlin Institute of Health Center for Regenerative Therapies, Charité - Universitätsmedizin Berlin, Berlin, Germany

**Keywords:** Coronary artery disease, Atherosclerosis, Cardiovascular disease, Biomarkers, Epigenetics, Angiography, LipidCardio study, Berlin Aging Study II, BASE-II

## Abstract

**Introduction:**

People age biologically at different rates. Epigenetic clock-derived DNA methylation age acceleration (DNAmAA) is among the most promising markers proposed to assess the interindividual differences in biological age. Further research is needed to evaluate the characteristics of the different epigenetic clock biomarkers available with respect to the health domains they reflect best.

**Methods:**

In this study, we have analyzed 779 participants of the LipidCardio study (mean chronological age 69.9 ± 11.0 years, 30.6% women) who underwent diagnostic angiography at the Charité University Hospital in Berlin, Germany. DNA methylation age (DNAm age) was measured by methylation-sensitive single nucleotide primer extension (MS-SNuPE) and calculated with the 7-CpG clock. We compared the biological age as assessed as DNAmAA of participants with an angiographically confirmed coronary artery disease (CAD, *n* = 554) with participants with lumen reduction of 50% or less (*n* = 90) and patients with a normal angiogram (*n* = 135).

**Results:**

Participants with a confirmed CAD had on average a 2.5-year higher DNAmAA than patients with a normal angiogram. This association did not persist after adjustment for sex in a logistic regression analysis. High-density lipoprotein, low-density lipoprotein, triglycerides, lipoprotein (a), estimated glomerular filtration rate, physical activity, BMI, alcohol consumption, and smoking were not associated with DNAmAA.

**Conclusion:**

The association between higher DNAmAA and angiographically confirmed CAD seems to be mainly driven by sex.

**Supplementary Information:**

The online version contains supplementary material available at 10.1186/s13148-023-01434-8.

## Background

Chronological age is closely associated with physical and cognitive capacity, morbidity, and mortality. Moreover, it is the most informative cardiovascular risk factor. Nevertheless, chronological age is unable to depict the remarkable heterogeneity of biological aging rates observed in same-aged peers. Further, the increasing life expectancy and the demographic shift towards a chronological older world population (UN Department of Economics and Social Affairs, 2019) foster the demand for clinically meaningful biomarkers of biological aging.

Due to its accuracy to predict chronological age and its association with morbidity and mortality [1], the epigenetic clock has been suggested as a promising biomarker of aging [2–4]. The epigenetic clock’s parameter, DNA methylation age (DNAmA), is calculated in years by the weighted DNA methylation fraction of a number of cysteine-phosphate-guanine (CpG) dinucleotides, which were selected by penalized regression analysis [2, 3, 5, 6]. Several different epigenetic clock algorithms are available that differ in number and location of CpG sites as well as in the outcome they were trained to predict [3, 5–7]. In this study, we analyzed DNAmA acceleration (DNAmAA), the deviation of DNAmA from chronological age, that was calculated with the 7-CpG clock [7]. This clock was previously shown to be associated with chronological age [7], vitamin D level [8, 9], diabetic complications [10], and cardiovascular health [11], but not with geriatric assessments, frailty [9, 12], lung capacity [13], and perceived psychological stress [14]. In contrast to other epigenetic clocks requiring high throughput equipment, the 7-CpG clock can be measured cost-effectively by methylation-sensitive single nucleotide primer extension (MS-SNuPE) [15] even in smaller laboratories. A different epigenetic clock algorithm which consisted of seven CpGs as well but differed in one CpG position from the algorithm employed here was previously analyzed in the LipidCardio cohort and was shown to be associated with chronological age and to be independent from relative telomere length [16].

Coronary artery disease (CAD) is a chronic cardiovascular disease, which is characterized by the progressive atherosclerotic plaque formation reducing the lumen of coronary arteries, eventually leading to chronic ischemia of the myocardium, chronic and acute coronary syndromes, heart failure or cardiovascular death. While improvements in health behavior (including balanced diet, physical activity, reduced smoking), efforts to manage cardiovascular risk factors (including hypertension and dyslipidemia), and advances in the medical treatment of coronary syndromes have reduced the incidence of CAD and CAD mortality, CAD remains the most common cause of death in Germany and globally [17–19].

The association of the epigenetic clock and CAD as well as its risk factors has recently been assessed by a number of studies, which, however, were yielding inconclusive results [20–28]. In the current study, we aim to test whether angiographically confirmed CAD is associated with increased DNAmAA, determined by the 7-CpG epigenetic clock.

## Methods

A detailed description of the LipidCardio study has been published previously [29]. In brief, patients aged 18 years and above, who underwent diagnostic cardiac catheterization for coronary angiography at the department of cardiology at Campus Benjamin Franklin university hospital, Charité – Universitätsmedizin Berlin, between October 2016 and March 2018, were eligible for inclusion, independent of their diagnosis and after providing written informed consent. Patients with acute troponin-positive coronary syndrome were not eligible for inclusion. Aim of this observational study was to collect patients’ clinical data to enable cross-sectional analyses focusing on CAD and its risk factors. The coronary angiography was performed by a trained cardiologist according to the standard protocol and documentation routine employed at the clinical unit. The results of the coronary angiography allowed the allocation of patients into three groups: (1) controls (non-pathological angiogram, i.e., without evidence of atherosclerosis), (2) non-obstructive atherosclerosis and CAD with a lumen reduction below or equal to 50%, and (3) clinical obstructive CAD with a lumen reduction exceeding 50%.

The LipidCardio study was conducted in accordance with the Declaration of Helsinki and approved by the ethics committee of the Charité-Universitätsmedizin Berlin, approval number EA1/135/16.

### Methylation-sensitive single nucleotide primer extension (MS-SNuPE)

Blood was drawn during the cardiac catheterization of the coronary angiography procedure, either via a peripheral intravenous access or from the radial or the femoral artery sheath post-heparin administration, for the purpose of routine laboratory testing, leukocyte DNA extraction, and biobank storage. Whole blood EDTA samples were frozen at − 80 °C following their collection to allow for a one-batch leukocyte DNA isolation with the sbeadex livestock kit in accordance with the manufacture’s protocol (LGC Genomics GmbH, Berlin Germany).

The methylation fraction of the CpG dinucleotide positions cg09809672, cg02228185, cg19761273, cg16386080, cg17471102, cg24768561, and cg25809905 (as well as cg10917602) was measured [7, 30, 31]. Briefly, 500 ng leukocyte DNA was bisulfite converted, according to the manufacture’s protocol of the EZ-96 DNA Methylation-Lightning Kit (ZYMO Research, Irvine, CA, USA). DNA was amplified by multiplex polymerase chain reaction (mPCR) and enzymatically cleaned, prior to and after undergoing single nucleotide primer extension (SNuPE). A negative control of HPLC water was run alongside the samples on each multiplex plate during the procedure and analyzed alike the samples in a 3730 DNA analyzer (Applied Biosystems, Waltham, MA, USA). GeneMapper software package 5 (Thermo Fisher Scientific, Waltham, MA, USA) was employed to assess the measurement quality and to determine peak height ratios to calculate the DNA methylation fraction at each CpG dinucleotide, as suggested by Kaminsky et al. [15]. Samples that showed a signal intensity below 200 at one or more CpG sites were excluded because of the “low peak height” definition by the manufacturer. A more extensive description of methods can be found in reference [16]. Although originally developed for MS-SNuPE, a newly available adjustment formula makes it possible to use the 7-CpG clock algorithm with methylation data obtained from Illumina’s MethylationEPIC array [32].

### DNAm age and DNAm age acceleration

DNAmA was estimated by the 7-CpG clock algorithm [7] which was trained on samples of the Berlin Aging Study II [33] to predict chronological age. This algorithm incorporates methylation information from following CpG sites: cg09809672, cg02228185, cg19761273, cg16386080, cg17471102, cg24768561, and cg25809905. DNAmAA was calculated as residuals of a linear regression analysis of DNAmA on chronological age. Chronological age was determined by subtracting the date of inclusion from the date of birth, divided by 365.25 days, taking leap years into account.

### Laboratory parameters and lifestyle variables

High-density lipoprotein (HDL), low-density lipoprotein (LDL), triglycerides, and lipoprotein (a) were measured in an accredited standard laboratory (Labor Berlin GmbH, Berlin, Germany). Blood pressure was measured on the right and left arm. The average of systolic blood pressure of both measurements was used in this study. Physical activity was assessed with the Rapid Assessment of Physical Activity (RAPA) [34] that relies on the participants self-reported levels of activity. Body mass index was calculated as body weight (in kilogram) divided by the squared body height (in meters). Estimated glomerular filtration rate (eGFR) was calculated by the equation developed by Cockcroft and Gault [35].

Coronary angiograms and methylation data were available for 779 participants of the LipidCardio study. The proportion of missing values was 20% or lower for all variables except for triglycerides which were derived from routine laboratory analyses conducted during the stay of the participants in the clinic. We do not believe that any systematic bias affected the probability that a certain value is missing. Therefore, we do not expect missing values substantially affecting our results.

### Statistical analysis

An available case analysis was performed. Participants for whom DNAmAA and CAD status were available were stratified according to their angiographically confirmed CAD status: (1) patients with normal angiogram, (2) non-obstructive CAD with an atherosclerotic lumen reduction below or equal to 50%, and (3) obstructive clinical CAD with a lumen reduction exceeding 50% for the purpose of the descriptive statistics and subsequent analyses. A univariate analysis of variance (ANOVA) was used to determine statistical significance of differences in DNAmAA and potential covariates between CAD groups. Post hoc contrast analyses were used to test for significance between specific groups. A Kruskal–Wallis test was employed to assess group differences of nominally scaled variables. Binary logistic regression analyses of CAD status (no CAD vs. obstructive CAD) on DNAmAA were performed to assess the association between CAD status and DNAmAA. Differences in DNAmAA between groups stratified by binary variables were assessed by two-sided t test for independent samples.

All statistical analyses were performed with the IBM SPSS Statistics software package, version 27. The figure was built with the R software package (version 4.2.0) [36] and the “ggplot2” package [37]. A p value below 0.05 was defined to be statistically significant.

## Results

### Study population

Data on DNAmAA and CAD status were available for 779 participants of the LipidCardio study. The mean chronological age was 69.9 ± 11.0 years, and 30.6% of all participants were women. Chronological age did not differ statistically significant between women (mean age: 70.7 ± 10.7 years) and men (mean age: 69.7 ± 11.1 years, *p* = 0.2, *t* test). Obstructive CAD with a lumen reduction exceeding 50% was confirmed angiographically in 71.1% of the participants (Table 1). 11.6% of the participants were diagnosed with non-obstructive CAD with an atherosclerotic lumen reduction below or equal 50%, while a normal angiogram was observed in 17.3% of the participants. Analysis of variance revealed statistically significant differences between CAD groups (no CAD vs. non-obstructive CAD vs. obstructive CAD) for HDL, LDL, lipoprotein (a), and diagnosed type 2 diabetes mellitus (*p* < 0.05, Table 1). No between-group differences were found for triglycerides, eGFR, BMI, physical activity, alcohol consumption, and current smoking status (Table 1). However, none of the continuously scaled variables were correlated with DNAmAA (Spearman’s *r* < 0.09, Additional file 1: Table S1). Similarly, no statistically significant differences were found in DNAmAA between groups stratified by alcohol consumption (yes: 0.06 years, no: 0.13 years, *p* = 0.9, *t* test), smoking (yes: 0.49 years, no: 0.05 years, *p* = 0.7, *t* test), and diagnosed type 2 diabetes mellitus (yes: 0.85 years, no: − 0.32 years, *p* = 0.2, *t* test). These potential confounding variables were therefore not included as confounders in the following regression analyses.

**Table 1:** Cohort characteristics. Statistical significance of differences between groups was assessed with ANOVA and Kruskal–Wallis test

	No CAD group (control group)					Non-obstructive CAD					Obstructive CAD					*p* value
	*n*	Mean, %	SD	Min	Max	*n*	Mean, %	SD	Min	Max	*n*	Mean, %	SD	Min	Max	
Chronological Age	135	64.79	12.52	21.28	85.17	90	70.41	9.34	42.91	89.59	554	71.17	10.48	34.22	91.22	< 0.001
DNAmA	135	64.85	12.93	30.52	134.84	90	69.40	13.36	27.83	139.14	554	72.00	14.22	29.43	138.66	< 0.001
DNAmAA	135	− 1.84	9.97	− 42.26	58.34	90	− 1.38	11.51	− 37.05	58.46	554	0.67	12.04	− 39.96	63.92	0.040
Systolic blood pressure (mmHg)	105	134.12	19.80	84.00	191.50	72	131.42	17.64	92.00	181.50	444	135.63	21.34	92.50	205.50	0.255
HDL (mg/dl)	125	56.69	18.32	22.00	102.00	82	55.27	17.91	26.00	114.00	537	48.83	15.36	14.00	108.00	< 0.001
LDL (mg/dl)	125	115.67	36.69	40.00	230.00	82	106.83	39.03	40.00	231.00	539	93.55	40.03	18.00	268.00	< 0.001
Triglycerides (mg/dl)	90	131.54	73.64	33.00	570.00	57	140.56	70.91	41.00	393.00	407	149.13	91.77	34.00	625.00	0.203
eGFR (ml/min/1.73m2)	114	75.34	28.83	24.98	163.81	82	81.94	28.00	4.81	165.30	495	80.30	29.86	9.31	221.51	0.205
Lipoprotein (a) (nmol/l)	133	40.64	53.23	5.00	240.20	88	45.29	62.57	5.00	256.30	542	62.00	82.22	5.00	478.20	0.005
BMI (kg/m2)	120	28.00	5.32	17.40	48.40	84	27.70	5.50	18.10	51.10	508	27.68	4.65	17.10	46.30	0.812
Physical activity (RAPA)	112	4.38	1.99	1	7	73	4.11	2.10	1	7	463	4.02	1.94	1	7	0.209
Alcohol consumption																
Yes	65	44.90				46	51.10				274	55.20				0.927
No	53	55.10				34	37.80				222	44.80				
Currently smoking																
Yes	24	20.10				14	16.90				95	18.20				0.826
No	94	79.90				69	83.10				409	21.80				
Diagnosed Type 2 Diabetes Mellitus																
Yes	26	19.30				20	22.20				166	30.00				0.023
No	109	80.7				70	77.80				388	70.00				
Sex																
Female	83	61.5				37	41.1				118	21.3				
Male	52	38.5				53	58.9				436	78.7				

*DNAmA* DNA methylation age, *DNAmAA* DNAmA acceleration, *HDL* high-density lipoprotein, *LDL* low-density lipoprotein, *eGFR* estimated glomerular filtration rate

### DNAm age acceleration and cardiovascular disease

The cohort’s mean DNAmAA, calculated as residuals of a linear regression analysis of DNAmA on chronological age, was 0.00 ± 11.7 years (*N* = 779).

**Fig. 1:**
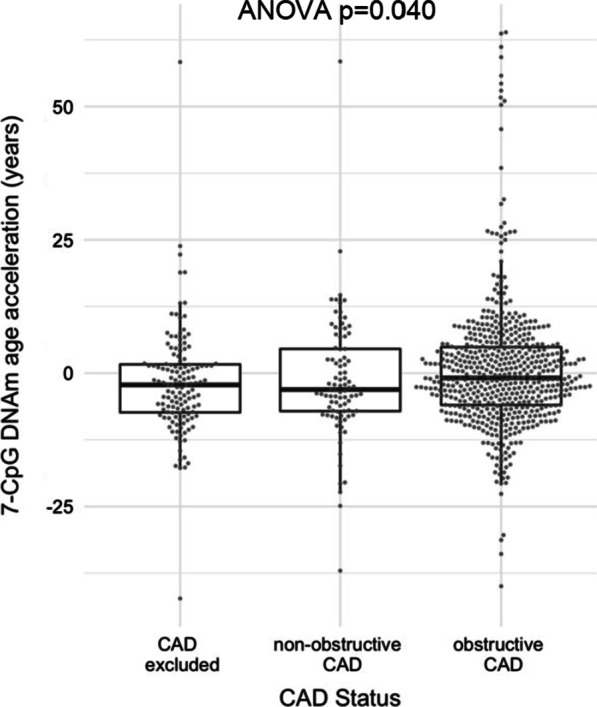
DNAmAA stratified by CAD status in participants of the LipidCardio study (*n* = 779). Statistical significance of difference between groups was assessed by ANOVA (*p* = 0.040) and post hoc contrast analyses. Mean 7-CpG DNAmAA differed statically significant between participants with no CAD and obstructive CAD (*p* = 0.025). Additionally, a statistically significant difference was found when comparing the mean of the combined group of participants with excluded CAD and with non-obstructive CAD with the group of participants with obstructive CAD (*p* = 0.015)

DNAmA correlated moderately with chronological age (Pearson’s *r* = 0.56). DNAmAA was on average 2.5 years higher in participants with an obstructive CAD compared to the control group with a normal angiogram (post hoc contrast analysis, *p* = 0.025, Fig. 1). Because no statistically significant difference in DNAmAA of patients with non-obstructive CAD and both other groups was found (post hoc contrast analyses, *p* > 0.12, Fig. 1), this group (*n* = 90) was excluded from the subsequent logistic regression analyses to create a better and more clearly defined control. In a logistic regression analysis of CAD status (no CAD vs. obstructive CAD) on DNAmAA, the association between CAD status and DNAmAA did not remain significant after adjustment for sex (Table 2). Similarly, sex-stratified subgroup analyses did not reveal any statistically significant association between DNAmAA and obstructive CAD (Table 2).

**Table 2:** Logistic regression analyses of CAD status (no CAD vs. obstructive CAD) on DNAmAA in 689 participants of the LipidCardio cohort

Model	Independent variable	OR	Lower 95% CI	Upper 95% CI	*p* value
Women and men					
1	DNAmAA	1.021	1.003	1.041	0.026
2	DNAmAA	1.012	0.994	1.031	0.191
	Sex (female)	0.175	0.117	0.262	< 0.001
Women					
	DNAmAA	1.002	0.979	1.026	0.851
Men					
	DNAmAA	1.027	0.997	1.057	0.08

*DNAmAA* DNA methylation age acceleration, *SE* standard error, *OR* odds ratio

## Discussion

In this study of 779 participants of the LipidCardio study who underwent diagnostic angiography, a 2.5-year higher 7-CpG DNAmAA was found in patients with obstructive CAD when compared with participants with a normal angiogram (*p* = 0.025). However, this association did not persist in logistic regression analyses adjusted for sex. In this study, men had a 3.1-year higher DNAmAA compared to women (*p* = 0.001). However, the higher DNAmA (compared to chronological age) in men is a frequently observed phenomenon [11, 12, 21, 38] for which the reasons are still unclear. However, this sex-dependent difference in DNAmA estimation might be the reason for a lack of statistical significance in our logistic regression analyses after adjustment for sex which is an import risk factor for CAD, as well. On the other hand, the lack of statistical significance in sex-stratified analyses could be due to a lack of power in these subgroup analyses.

Although numerous studies analyzed the association between cardiovascular health (CVH)-associated phenotypes and biological age, this study is the first to examine differences in DNAmAA with respect to angiographically confirmed CAD. The missing association between DNAmAA and CAD after adjustment for sex reported here is in line with results by Horvath and colleagues [21]. No association between Horvath’s DNAmAA and incident CHD was present in 1462 women (mean age: 63 years, age range: 50–80 years) of the WHI study. Similarly, no association between Horvath or Hannum clock estimates and CVD-associated mortality was reported by Dugue and colleagues in a cohort of 2818 participants (age: 59.0 ± 7.6, 49% women) [20]. In the same vein, no association between Hannum or Horvath Δ_age_ and cardiovascular disease was found in participants of the LBC1921, LBC1936, and NAS cohorts (mean age between 69.5 and 79.1 years, between 0 and 60% women) [39]. In this study, only when analyzing participants of the FHS cohort a weak but statistically significant association was found (Hannum: *β*⁠ = − 0.13, SE = 0.06, *p* = 0.02, Horvath: *β*⁠ = − 0.27, SE = 0.06, *p* = 6.9 × 10^–6^) [39].

In contrast to the reports mentioned above and the results described in this study, Perna and colleagues found a 20% increase in risk for cardiovascular mortality for every 5-year increase in Horvath DNAmAA (but no association with Hannum DNAmAA was found) [23]. Similarly, Lind and colleagues found a statistically significant association between Horvath DNAmAA and the risk to develop a CVD during a 10-year follow-up period but not for Hannum DNAmAA [22]. Further, associations between Horvath and Hannum DNAmAA and fatal CAD outcomes were reported by Roetker and colleagues [24].

Recent studies analyzed second-generation (e.g., PhenoAge [6] and GrimAge [5]) in addition to first-generation clocks (e.g., Horvath, Hannum, 7-CpG clock). In contrast to first-generation clocks that were trained to predict chronological age, second-generation clocks employ more complex composite markers to select and weigh the CpG positions of their algorithms. Potentially due to this more inclusive approach, second-generation clocks were more frequently reported to be associated with disease than first-generation clocks. Lo and Lin analyzed 2474 participants of the Taiwan Biobank (mean age: 49.8 years, SD: 11.1 years) in context of their cardiovascular health. After the exclusion of participants with CVD, the PhenoAge and GrimAge DNAmAA but not the Horvath or Hannum DNAmAA were associated with a CVH score [25]. A similar finding was reported by Joyce and colleagues who reported an association between Life’s Simple Seven (LS7) and GrimAge DNAmAA in the CARDIA study and FHS cohort. The DNAmAA derived from the Horvath clock showed similar but weaker associations and the Hannum DNAmAA was not associated with the LS7 [40]. Although an exhaustive analysis of the literature is beyond the scope of this manuscript, the described results suggest that second-generation clocks might be more sensitive to cardiovascular health. It is, however, important to stress that results vary widely, and the comparability between studies is limited due to the in part substantial differences in assessment and selection of covariates, demographics, and sex distribution of the analyzed cohorts as well as the epigenetic clock algorithms employed. Furthermore, the phenotypes and variables used to assess the participants’ cardiovascular health differ greatly. Potentially a substantial part of the differences between the findings described above can be attributed to these differences between cardiovascular outcome variables. As our understanding of the underlying mechanisms that lead to biological age-associated changes in the epigenome and its relationship to health- and age-associated variables is still very limited, further studies are needed to clarify the potential use of epigenetic clock derivatives with respect to specific aspects of cardiovascular health in the clinical context.

We want to point out several limitations to this study. First, because methylation data were measured with the MS-SNuPE method, only the 7-CpG epigenetic clock, but no other clock algorithm, was available for analysis. However, the 7-CpG clock was validated in several cohorts [7, 41] and analyzed with respect to different age-associated phenotypes [10, 12, 14]. Furthermore, the MS-SNuPE method used to measure 7-CpG DNAmA seems to provide results that are comparable to those obtained through Illumina’s MethylationEPIC array [32]. Second, although it is difficult to estimate its impact, we expect a selection bias in our cohort. All participants underwent a diagnostic angiography, and therefore, no healthy control group was available. However, participants with no apparent CAD had less chronic disease and reported less risk factors for CAD. A detailed description of the cohort and risk factors with respect to apparent CAD can be found in ref. [29]. Third, most participants had an obstructive CAD which results in a comparatively small sample size for the control group.

Strengths of this study include the comparatively large sample size of a well-characterized cohort. Additionally, this is to our knowledge the first study that makes use of angiographic data in the context of epigenetic clocks, allowing a particularly reliable classification into the groups that were compared.

## Conclusion

In conclusion, patients with angiographically confirmed CAD were epigenetically 2.5 years older compared to patients with a normal angiogram. However, in logistic regression analyses DNAmAA was not associated with CAD after adjustment for sex.

## Supplementary Information

**Additional file 1**. **Supplementary Table 1.** Correlation table of DNAmAA and potentially confounding variables (Spearman’s r).

## Data Availability

The datasets used and/or analyzed during the current study are available upon reasonable request.
