# Supplementary material for: Epigenetic aging in patients diagnosed with coronary artery disease: results of the LipidCardio study
Source: Clin Epigenetics. 2023 Jan 31;15:16. doi: 10.1186/s13148-023-01434-8 (PMC9887837; doi:10.1186/s13148-023-01434-8)
Supplement: Supplementary file 1 — Additional file 1. Supplementary Table 1. Correlation table of DNAmAA and potentially confounding variables (Spearman’s r). [file 13148_2023_1434_MOESM1_ESM.docx]

Supplementary Table 1: Correlation table of DNAmAA and potentially confounding variables (Spearman’s r).

|  |  | DNAmAA (years) |
| --- | --- | --- |
| Systolic blood pressure (mmHg) | r | -0.07 |
|  | p-value | 0.082 |
|  | n | 621 |
| HDL (mg/dl) | r | -0.019 |
|  | p-value | 0.596 |
|  | n | 744 |
| LDL (mg/dl) | r | -0.008 |
|  | p-value | 0.821 |
|  | n | 746 |
| Triglycerides (mg/dl) | r | 0.032 |
|  | p-value | 0.458 |
|  | n | 554 |
| eGFR (ml/min/1.73m2) | r | 0.004 |
|  | p-value | 0.908 |
|  | n | 691 |
| Lipoprotein (a) (nmol/l) | r | 0.005 |
|  | p-value | 0.884 |
|  | n | 763 |
| BMI (kg/m2) | r | -0.01 |
|  | p-value | 0.793 |
|  | n | 712 |
| Physical activity (RAPA) | r | -0.008 |
|  | p-value | 0.832 |
|  | n | 648 |
